# Alkaline phosphatase–streptavidin conjugate (APSA) enzyme and binding activity over time and storage conditions

**DOI:** 10.1016/j.bbrep.2025.102160

**Published:** 2025-07-21

**Authors:** Nan Cheng, Lloyd Johnson, Jaimie Dufresne, Sina Mazinani, John G. Marshall

**Affiliations:** Research Analytical Biochemistry Laboratory, Department of Chemistry and Biology, Faculty of Science, Toronto Metropolitan University, 350 Victoria Street, Toronto, Ontario, M5B 2K3, Canada

**Keywords:** APSA, alkaline phosphatase streptavidin conjugate, Storage conditions, Freezing, freeze drying, Protease inhibitors, Glycerol, sucrose, trehalose, BCIP/NBT

## Abstract

Alkaline phosphatase (AP) linked to streptavidin (SA) in the form of the APSA enzyme conjugate is required for diagnostic screening for a variety of clinical conditions world wide. The enzyme activity of APSA conjugates in the liquid phase showed variation across samples that declined with storage time. Random sampling of the enzyme activity in the liquid phase (ANOVA p < 2E-16; Regression p < 0.043) and the binding plus enzyme activity of APSA in the model assay (R^2^ > 0.99) of biotinylated human IgG (B-h-IgG) directly adsorbed to 96 well plates showed a similar loss of function over time (ANOVA p < 9.15E-15, Regression p <1.1E-9). The enzyme AP showed little dissociation from the SA moiety while proteolysis of the BSA carrier was observed. Covalent protease inhibitors 4-(2-aminoethyl)benzenesulfonyl fluoride hydrochloride (AEBSF) or tosyl-l-lysine chloromethyl ketone hydrochloride (TLCK) abrogated AP enzyme activity, but the competitive inhibitors epsilon-aminocaproic acid (EACA) and benzamidine (BNZ) had no protective effect on APSA activity over time. Samples of APSA showed large variation in enzyme activity (p ≤ 2E-16) and so were titrated by the colorimetric assay and standardized against indigo blue in DMSO to achieve an initial OD value of ∼1.0 at 595 nm prior to following activity over storage time. After titration, the effect of temperature, addition of glycerol prior to freezing, and freeze drying with or without trehalose and sucrose, on alkaline phosphatase activity was compared using a sampling schedule over storage time. The alkaline phosphate activity was not immediately sensitive to freeze-drying but was sensitive to storage time and ultra-low temperatures, but the addition of sugars or glycerol to the APSA prevented some of the activity loss. Storage of APSA on wet ice or in 50 % glycerol at −20 °C retained about 50 % of the starting optical density reading of APSA after 170 days in storage.

## Highlights

• Freeze drying in sucrose and trehalose preserves APSA activity.
•Tryptic or chymotryptic proteolysis is not the mechanism of APSA degradation.
•Storage on wet ice or in 50 % glycerol at −20 °C best preserves APSA.
•Ultra freezing or cryofreezing is deleterious to APSA.
•The half life of APSA activity in 50 % glycerol at −20 °C is 170 days.

## Graphical abstract

**Figure:**
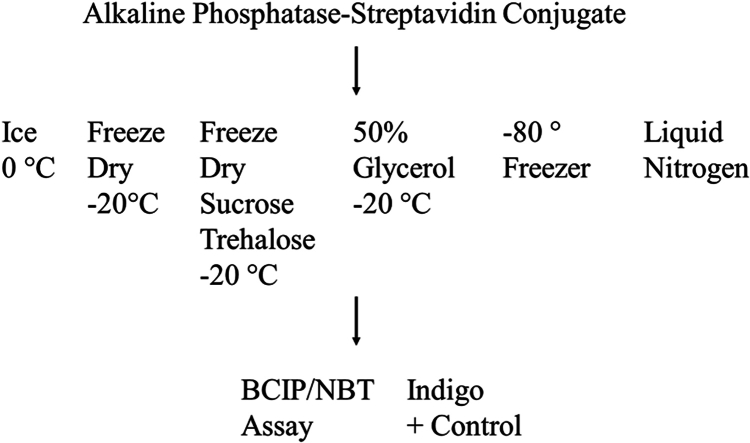

## Abbreviations

AEBSF: 4-(2-aminoethyl)benzenesulfonyl fluoride hydrochloride
APSA: alkaline phosphatase streptavidin conjugate
Blue Phos: liquid BCIP/NBT substrate surfactant
BNZ: Benzamidine hydrochloride
DF: dilution factor
DMSO: dimethyl sulfoxide
EACA: Epsilon-aminocaproic acid
h-IgG: human IgG
TLCK: Tosyl-l-lysine chloromethyl ketone hydrochloride

## 1 Introduction

Alkaline phosphatase (AP) cross linked with glutaraldehyde to streptavidin (SA) is a vitally important enzyme conjugate (APSA) that serves as a universal signal amplification reagent in many biological assays, is widely used and has great industrial importance in diagnostic testing [[1], [2], [3]]. APSA enzyme conjugate is frequently used in screening with diagnostic reagents both in laboratories and in point of care under a variety of environmental conditions. Despite its great importance there is paucity of peer reviewed and published data regarding the half-life of the APSA conjugate under different cold storage regimes. The change in APSA activity over time may be a confounding source of error in the reported detection limits of ELISA and hybridization assays. Alkaline phosphatase is a nearly perfect enzyme [4] for signal amplification, that can hydrolyze a wide variety of substrates containing phosphate esters [[5], [6], [7]]. Streptavidin shows high affinity for its binding partner biotin with a *K*_d_ of ∼E-14 [8]. Thus, the conjugate of the enzyme alkaline phosphatase (AP) covalently bonded to streptavidin (SA) by glutaraldehyde shows high enzyme activity and the high binding affinity to detect biotinylated molecules such as proteins, antibodies and nucleic acids in ELISA, immunohistochemistry, Western blot and hybridization assays [[9], [10], [11], [12]]. The indigo blue dye product of alkaline phosphatase reacted with BCIP/NBT (5-bromo-4-chloro-3-indolyl-phosphate/nitro blue tetrazolium) is poorly soluble in water but will dissolve efficiently in 1 % Tween 20 in aqueous assays to characterize alkaline phosphatase activity in a 96 well liquid format [13]. The signal amplification by APSA can be detected by colorimetric, fluorescence or mass spectrometric methods [14].

The mechanisms that lead to the loss of APSA enzyme conjugate function over time affect the sensitivity (i.e. detection limits) and reproducibility of assays with low detection limits soon after conjugation, showing a trend to the loss of sensitivity over time**,** and thus APSA needs to be frequently assessed for quality control purposes. With a steady increase in the sensitivity of the detection methods [15] and a growing demand for assay sensitivity and stability, an understanding of how storage conditions affect APSA's activity over time may become an important factor in use of APSA in diagnostic and analytical assays. While the total function of the APSA conjugate declines over time, it is not immediately apparent if the decline results from the loss of alkaline phosphatase enzyme activity and/or the loss of biotin binding capability from the cross-linked streptavidin or proteolytic degradation. The addition of albumin has been shown to extend the activity of alkaline phosphatase [16]. The use of an indigo blue absolute standard curve may ensure the validity of comparing assays performed on different days and months.

The loss of APSA function may confound the measurement of detection limits in binding assays and may have profound implications for assay development and screening binding reagents against immobilized antigens [[17], [18], [19]]. There have been several studies on the stability and the half-life of alkaline phosphatase under room temperature and heat stress conditions with the use of polyols [20] but there is no comprehensive side-by-side comparison of different storage methods to best preserve the APSA conjugate. Despite its importance and ubiquity, there are no studies that have systematically examined APSA conjugate inhibitor and cold storage regimes [16,[20], [21], [22], [23]] for their effect on enzyme and its binding activity over time [24]. While several studies have examined the stabilization of the alkaline phosphatase (AP) enzyme itself at elevated temperature, there are no studies on the stability of the conjugate under cold versus frozen or freeze-dried storage conditions. It is important to understand the conditions that will best preserve the alkaline phosphatase conjugates for diagnostic and biochemical assays.

Herein human IgG (hIgG) was used as a model antigen to measure APSA enzyme's activity and binding. Electrophoresis by SDS-PAGE with Coomassie brilliant blue R250 (CBBR) staining and native PAGE with the Western blot were used to examine the structural and proteolytic stability of the APSA conjugate. The effect of different protease inhibitors on APSA activity was examined over time by the liquid BCIP/NBT assay. The BCIP/NBT reaction was also used to study how different storage conditions, including wet ice in the fridge (0–4 °C), a range of freezing temperatures, addition of glycerol, and drying by lyophilization with or without sugars, affect enzyme activity over a period of 170 days alongside the reference standard indigo blue.

## 2 Experimental methods

### 2.1 Materials

Alkaline phosphatase – streptavidin conjugate (APSA) was from Jackson Immuno Research Laboratories (West Grove, PA, USA) or Invitrogen. Jackson APSA was supplied at 1.0 mg/mL alkaline phosphatase (AP) purified from calf intestine coupled with streptavidin (SA) by glutaraldehyde in 0.01 M Tris-HCl, 0.25 M NaCl, pH 8.0 with 30 mg/mL bovine serum albumin (BSA, IgG-Free, Protease-Free) in 0.05 % sodium azide. Invitrogen alkaline phosphatase (AP) conjugate of streptavidin (Cat. no. S921) was supplied in 500 μL of a 2 mg/mL solution in 30 mM triethanolamine (TEA), 3 M NaCl, 1 mM MgCl_2_, 0.1 mM ZnCl_2_, pH 7.6, containing 1 % bovine serum albumin (BSA) and 2 mM sodium azide. Sucrose, glycerol, D-(+)-trehalose dehydrate, tris(hydroxymethyl)aminomethane (tris), Indigo Blue, dimethyl sulphoxide (DMSO), *N*α-Tosyl-l-lysine chloromethyl ketone hydrochloride (TLCK), 4-benzenesulfonyl fluoride hydrochloride (AEBSF), epsilon-aminocaproic acid (EACA) and benzamidine (BNZ) were obtained from Sigma-Aldrich (St. Louis, MO, USA). PVDF membranes (2 μm pore size) was purchased from EMD Millipore (Burlington, MA USA). Nunc™ Maxisorp™ 96 well polystyrene plates and EZ-Link NHS-PEG12-Biotin were obtained from Thermo Fisher Scientific (Waltham, MA, USA). For liquid phase reactions in 96 well plates the BCIP/NBT enzyme substrate in surfactant KPL BluePhos®Microwell Phosphatase Substrate System was obtained from SeraCare (Mandel, Guelph, ON, CA). For solid phase reactions on PVDF the 1-Step™ NBT/BCIP Substrate Solution was obtained from Sigma Aldrich (St. Louis, MO, USA). All the chemicals used in this study were of reagent grade or better without further purification unless otherwise mentioned. The 96 well plate reader was from Bio-Rad Laboratories (Hercules, CA, USA).

### 2.2 Liquid phase alkaline phosphatase colorimetric activity

One hundred microlitres (100 μL) of the APSA dilutions were reacted with 100 μL of the prepared phosphatase substrate solution in each well of a Nunc™ Maxisorp™ 96 well polystyrene plate. The BluePhos (BCIP/NBT) reagent solution was prepared according to the manufacturer's protocol. The KPL BluePhos Phosphatase Solution A and Solution B were warmed to room temperature. An equal volume of Solution A and Solution B were mixed in a centrifuge tube immediately before use. A 100 μL of the pre-warmed BluePhos Solution A plus B were added and incubated at 37 °C with agitation at 250 rpm for 30min before quantification on a Nunc microplate and read at 595 nm with the Bio-Rad 96 well plate reader.

### 2.3 Solid phase PVDF colorimetric APSA binding to biotinylated human IgG (B-h-IgG)

Human IgG was biotinylated at 20:1 molar ratio with EZ-Link NHS-PEG12-Biotin which contains the polyethylene glycol spacer arm. The biotinylated human IgG (B-h-IgG) was spotted onto PVDF that was pre-wet with methanol immediately before application and dried until opaque white. The membrane strip was blocked with 1 % IgG-Free BSA in 0.1 % deoxycholate prior to incubation with 5 mL of 1/5000 APSA immediately after receipt of the conjugate and after storage as indicated by the manufacturer.

### 2.4 Solid phase polystyrene biotinylated IgG biding and quantification

In order to determine the effect of storage on the APSA conjugates binding to biotin and alkaline phosphatase activity together, 10 ng B-h-IgG was used to coat wells of a Nunc™ Maxisorp™ 96 well polystyrene plate prior to blocking with 1 % BSA in the Tris pH 8.85 buffer and reacted with the APSA conjugate.

### 2.5 SDS-PAGE and native PAGE western blot against streptavidin

To determine the effect of storage on the structural integrity of streptavidin (SA) bonded to alkaline phosphatase (AP) over storage time, the APSA conjugate was resolved by 4–9 % gradient tris glycine SDS-PAGE [25] and stained by 0.1 % CBBR in 40 % methanol and 5 % acetic acid to observe the protein degradation profile. In addition, the APSA separated by Native PAGE without SDS was transferred to PVDF membranes and then the streptavidin sub-unit of the immobilized APSA was detected with biotinylated HRP (B-HRP) using enhanced chemiluminescence with the Bio-Rad imaging work station [26].

### 2.6 Covalent inhibitors of trypsin and chymotrypsin

The covalent inhibitors of serine proteases TLCK and AEBSF were tested for their effect on APSA activity. A 10 mM TLCK (Nα-Tosyl-l-lysine chloromethyl ketone hydrochloride) stock was freshly prepared in 1 mM HCl. A 10 mM AEBSF (4-benzenesulfonyl fluoride hydrochloride) stock was freshly prepared in water. The APSA conjugate provided by the manufacturer was diluted 1 in 10 (DF10) and then 200 μL was treated with 2 μL of the covalent inhibitors. The untreated APSA enzyme and the treated enzyme conjugates (APSA/TLCK and APSA/AEBSF) were used to created dilution series at 0, 10, 100, 250, 500, 750, 1000 pg/μL and 100 μL added to a 96 well plate before adding pre-warmed BluePhos Solution A and B mixture and incubated at 37 °C with agitation 30min.

### 2.7 Short term storage with competitive inhibitors of trypsin and chymotrypsin

Ten microliters (10 μL) of APSA dilutions from 5 to 150 pg/μL per well was immobilized into a 96 well plate (Millipore Sigma) immediately before use to achieve 50–1500 pg per well of immobilized APSA. 100 μL of 10 mM epsilon-aminocaproic acid (EACA) and benzamidine (BNZ) was added to each well before the immobilized APSA was incubated at 37 °C for 30 min in the presence of the inhibitors with or without washing prior to measurement of enzyme activity. Untreated APSA was served as the control. For APSA in the presence of the inhibitors with washing, the wells were washed six times with 200 μL of 20 mM Tris pH 8.85. A 150 μL volume of the pre-warmed BluePhos Solution A and B were added to each well and incubated at 37 °C with agitation in a 96 well microplate and read at 595 nm. APSA in the presence of 10 mM EACA, 10 mM BNZ, or 10 mM EACA plus 10 mM BNZ was incubated on ice, on the bench at RT or at 37 °C over 7 days for measurement of enzyme activity. Untreated APSA on ice served as the control. A 150 μL of the pre-warmed BluePhos Solution A and B were added to each well and incubated at 37 °C for 30min with agitation to the PVDF and the PS microplate and read at 595 nm.

### 2.8 Titration of APSA enzyme activity

Three independent samples of APSA were titrated to achieve an OD 595 nm of 1.0 versus the Indigo standard. Upon arrival, the fresh APSA solution was diluted over a series of concentrations from 5 to 5000 pg/mL with sterile 10 mM Tris HCl buffer, pH 8.85, to determine the dilution factor (DF) that resulted in an OD reading of 1.0 at day zero. The APSA dilution where the OD of the freshly arrived APSA equalled 1.0 was the working concentration for APSA samples in the scheduled time course study.

### 2.9 Indigo blue dye standard curve

A 10 mM stock of Indigo Blue was created in DMSO and used to make a dilution curve in 10 % DMSO curve from 1 to 1000 μM where 200uL was measured in each well. Regression analysis was used to indicate that 200 μL of 840 μM Indigo dissolved in 10 % DMSO provides an OD reading of 1.0 at 595 nm to standardize absorbance recordings over time. Alternatively indigo blue was dissolved in a reaction buffer with a Tween 20 as an analytical control to standardize alkaline phosphatase activity [13] where indicated.

### 2.10 Scheduled long term storage condition study

At each time shown, the APSA was diluted from the storage conditions to the previously determined working concentration (OD 595 nm = 1.0) prior to mixing 100 μL of the diluted APSA with 100 μL of the phosphatase substrate buffer and the microplate was incubated at 37 °C with agitation for 30min (n = 4) before reading the plate at 595 nm. Six different APSA storage conditions were studied: manufacturers stock solution on wet ice in a refrigerator (0 °C); mixed with an equal volume of glycerol and stored at −20 °C; lyophilized and stored at −20 °C; made to 1 M of sucrose and trehalose, lyophilized and stored at −20 °C; stocks at −80 °C; stocks in liquid nitrogen. Fresh upon arrival, four independent APSA samples were titrated to determine the dilution factor (DF) to reach the OD of 1.0 at 595 nm. The aliquots and the original stocks were stored for about 25-week (170 days) under the different cold conditions and sampled on a time schedule. The APSA enzyme activity under six different storage conditions was subsequently compared over a period of about 170 days by reaction with Blue Phos. For aliquots stored on wet ice, the undiluted APSA solution was kept in its original vial sealed with parafilm in a fridge. For the glycerol stock, the APSA solution was mixed with glycerol at 1: 1 v/v ratio to a final concentration of 50 %, and then stored at −20 °C. For lyophilization without additive, a 10 μL aliquot of APSA was transferred to a 1.7 mL centrifuge tube, lyophilized and then stored at −20 °C. Sucrose and trehalose were dissolved into water at a final concentration of 2 M each and then sterilized. For lyophilization with the sugar addition, the APSA solution was mixed with sterile 2 M sucrose/trehalose solution at 1: 1 v/v ratio, lyophilized and then stored at −20 °C. For low temperature freezing 10 μL per aliquot of the original APSA solution was transferred to a 1.7 mL tube or 2 mL cryovial, and stored in a −80 °C and in liquid nitrogen respectively. The APSA aliquots were removed from storage and maintained on ice during assay and then discarded after a single use. An independent sample of APSA with 2 mM sodium azide and 3 M NaCl was stored on wet ice in the refrigerator or in 50 % glycerol at −20 °C. For the time course study of the APSA activity at different storage conditions, one aliquot of APSA from each storage condition was used for each time point studied.

## 3 Results

### 3.1 Solid phase (PVDF) assay of biotinylated human IgG (B-h-IgG) assay over time

**Fig. 1:**
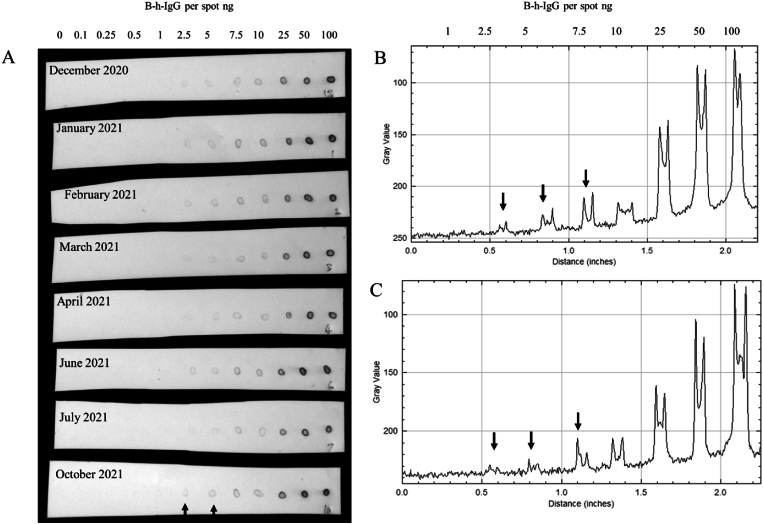
Random sampling of alkaline phosphatase streptavidin (APSA) with different storage duration on wet ice in the fridge (0–4 °C) by detection of biotinylated human IgG (B-h-IgG) dot blots on PVDF. Panels: A, Image of B-h-IgG dot blots on PVDF membranes and detected by APSA using the BCIP/NBT assay; B, image analysis of B-h-IgG samples on PVDF test strips reacted with fresh APSA; C, image analysis of B-h-IgG on PVDF test strips with APSA stored on wet ice for 22 months. Note the APSA conjugate retains the capacity to bind the B-h-IgG target on the solid phase over time yielding a circular pattern. All sampled reacted alongside the fresh sample from October 2021.

A dot blot of a biotinylated human IgG standard (B-h-IgG) dot blot on PVDF, similar in principle to a home testing strip, reached as low as 2.5 ng B-h-IgG but was robustly detected on dot blots at 10 ng of B-h-IgG by the BCIP/NBT reaction over 22 months of storage [27]. The BCIP/NBT colorimetric reaction produces the insoluble indigo blue dye product [13] for solid phase PVDF assays. In the solid phase assay the blue dye product is adsorbed to the hydrophobic PVDF at the site of the enzyme activity to form a visible circle about 1 mm in diameter [28] on the membrane surface (Fig. 1). The solid phase assay was frequently sensitive to 2.5 ng of B-h-IgG per spot upon first receipt of the APSA. However, over the course of weeks to months on ice the sensitivity was sporadically lost in some samples and it was sometimes challenging to reach detection of 2.5 ng of the B-h-IgG standard. The APSA conjugate retained the capacity to bind the B-h-IgG circular spot presented on PVDF when incubated with an excess of APSA in solution over the course of 22 months of storage on ice. The robust visible detection limit of the APSA on PVDF dot blots reached at least as low at ∼10 ng per spot after extended storage. We observed that the APSA sample batch and the storage time had a clear effect on the detection limit of B-h-IgG by dot blots where an excess of APSA was employed in the development buffer.

### 3.2 Random sampling of variation over samples and time

**Fig. 2:**
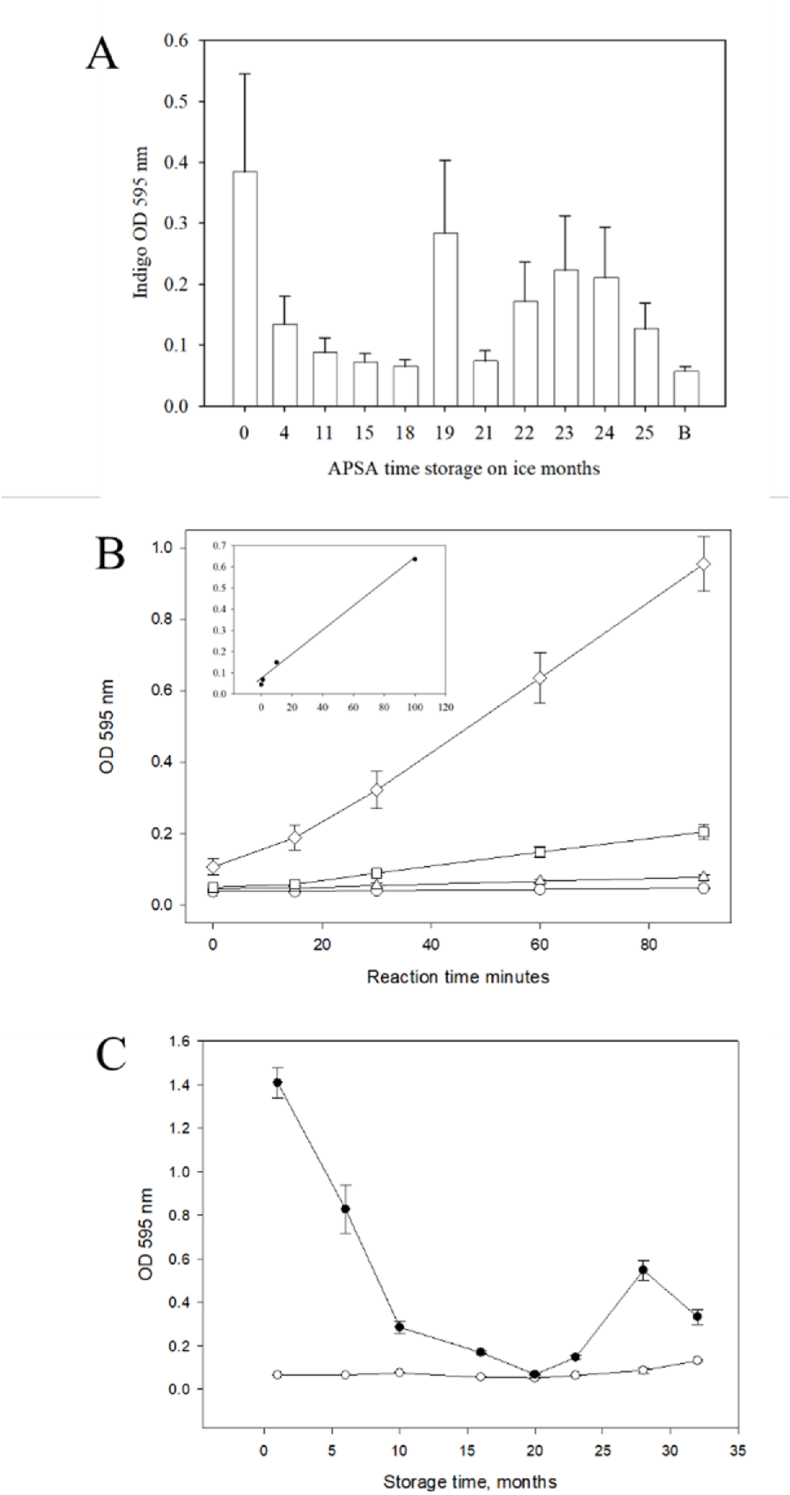
Random sampling of the APSA sample vials for the liquid phase enzyme activity and the solid phase binding activity to biotinylated human IgG (B-h-IgG) in 96 well plates. Panels: A, the sample variation in alkaline phosphatase activity of APSA conjugates measured over time per unit volume at a dilution factor (DF) of 1 in 250,000 (DF 250,000) in the liquid phase enzyme assay in 96 well plates (ANOVA p < 2E-16, Regression Intercept p ≤ 2.49E-10, p < 0.0435); B, the solid phase B-h-IgG assay in 96 well polystyrene plates reacted for 90 min with BluePhos substrate and measurement of optical density (OD) at 595 nm; Symbols: (○) 0 ng B-h-IgG; (△)1 ng B-h-IgG; (□) 10 ng B-h-IgG; (◇) 100 ng B-h-IgG (R**^2^** = 0.99, p-value: 0.002483**)** [Inset, linearity of APSA reaction from the 0, 1, 10 &100 ng per well]; C, APSA solid phase binding assay to B-h-IgG (100 ng per well) over storage time measured with stored on ice within a 4 °C refrigerator were compared to show the effect of sample to sample variation and storage time on both B-h-IgG binding activity and APSA catalytic activity. Symbols: (○) 0 ng B-h-IgG; (•) 100 ng B-h-IgG (ANOVA p < 9.15 E -15, Regression log months, R^2^: 0.7048, F-statistic: 75.01 on 1 and 30 DF, p-value <1.1E-9).

In contrast to B-h-IgG dot blots in the solid phase, APSA conjugate alone was dissolved in the liquid phase for assays in a 96 well plate alongside the indigo blue standard that may be quantified by the UV/VIS spectroscopy. Vials of APSA stored on ice in the fridge for up to 22 months and then sampled at a dilution factor of 1/250,000 (DF 250,000) prior to assay showed extreme sample to sample variation in enzyme activity and a trend to lower OD values over time with a highly significant effect of the storage time on the detection limit (ANOVA p ≤ 2E-16, Regression p < 0.043) (Fig. 2A). To quantify sensitivity of the APSA conjugate by random samples over time a quantitative liquid phase assay against biotinylated human IgG (B-h-IgG) immobilized in 96 well plates was constructed. Detection of the bound B-h-IgG from 1 to 100 ng on 96 well plates alongside blank controls showed that the biotinylated immunoglobulin might serve as a model system to examine detection limits of the half sandwich assay over time at the low nano gram range. The detection of B-h-IgG was resolved from the blank by 10 ng of B-h-IgG in 96 well plates from the production of indigo blue monitored at 595 nm (Fig. 2B). However, the sensitivity of the APSA detection of 100 ng B-h-IgG appeared to vary over samples and declined over and time in storage in 96 well plate assays (ANOVA 9.15E-15, Regression p < 1.1E-9) with a pattern similar to liquid phase enzyme assays (Fig. 2C).

### 3.3 Structural stability of APSA enzyme conjugate preparation over time

**Fig. 3:**
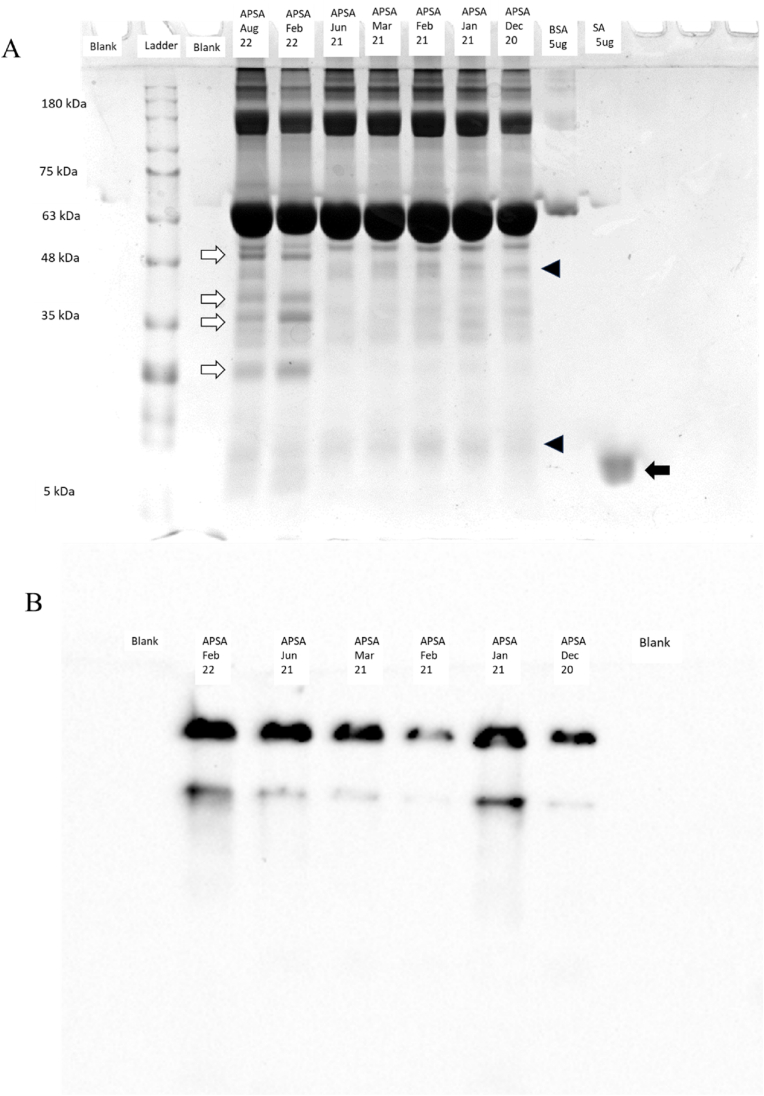
Analysis of the APSA conjugate by polyacrylamide gel electrophoresis. Panels: A, SDS-PAGE and Coomasie blue staining of the APSA conjugate stored on ice over time (Bands that fade, open arrow; Bands that appear black triangle; solid arrow, streptavidin); B, Native PAGE of APSA conjugate for Western blot with HRP-SA followed by ECL detection.

Electrophoresis by SDS-PAGE and native PAGE with the Western blot showed there is apparently proteolytic breakdown of the APSA preparation over time but that the streptavidin cross linked to the high molecular mass conjugate appeared stable with storage over time. Boiling samples in detergent prior to SDS-PAGE with Coomassie blue staining indicated that some apparent proteolytic degradation, at least for the BSA carrier protein, had occurred resulting in the fading or loss of some upper protein bands with the appearance of new lower bands over time (Fig. 3A). In order to determine if the apparent loss of B-h-IgG binding and/or enzyme activity was a result of degradation of the glutaraldehyde conjugate over time, we examined APSA conjugate by both denaturing SDS-PAGE and non-denaturing native PAGE. Native PAGE in the absence of detergent followed by the Western blot to PVDF with detection by biotinylated HPR showed that the majority of streptavidin was in a high molecular mass complex (Fig. 3B). However, some apparent fragments of the high molecular mass APSA complex were visible from anti streptavidin immunoblots.

### 3.4 Proteolytic stability of APSA

**Fig. 4:**
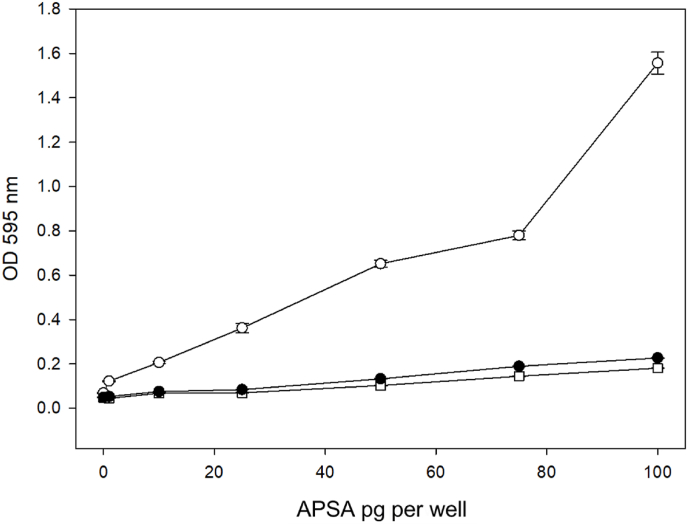
Effect of protease TLCK and AEBSF inhibitors on alkaline phosphatase activity on 96 well polystyrene plates. Untreated enzyme conjugate (○), TLCK treated (•) and AEBSF treated (□). Mean (n = 4) plus standard error shown.

**Fig. 5:**
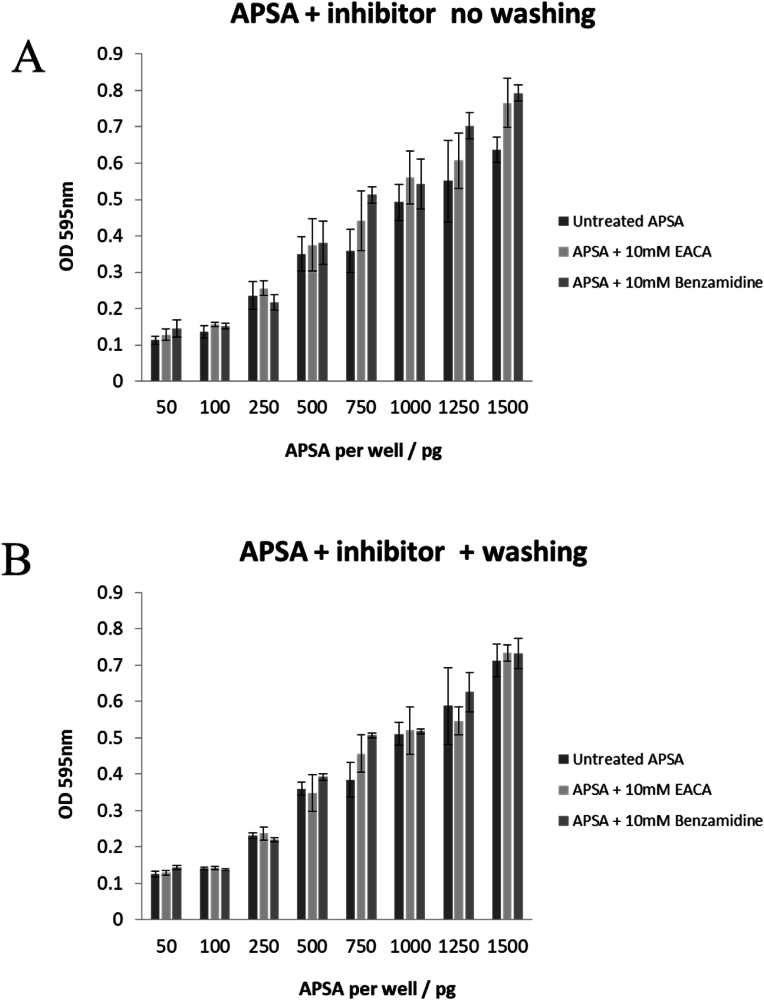
Effect of binding and/or washing of competitive protease inhibitor epsilon-aminocaproic acid (EACA) and benzamidine (BNZ) on immobilized APSA activity. Panels: A, immobilized APSA enzyme activity measured in the presence of competitive protease inhibitors; B, immobilized APSA enzyme activity measured after washing away competitive protease inhibitors. Mean (n = 4) plus standard error shown. The OD 595 nm is shown after correction against the blank (0 pg APSA).

**Fig. 6:**
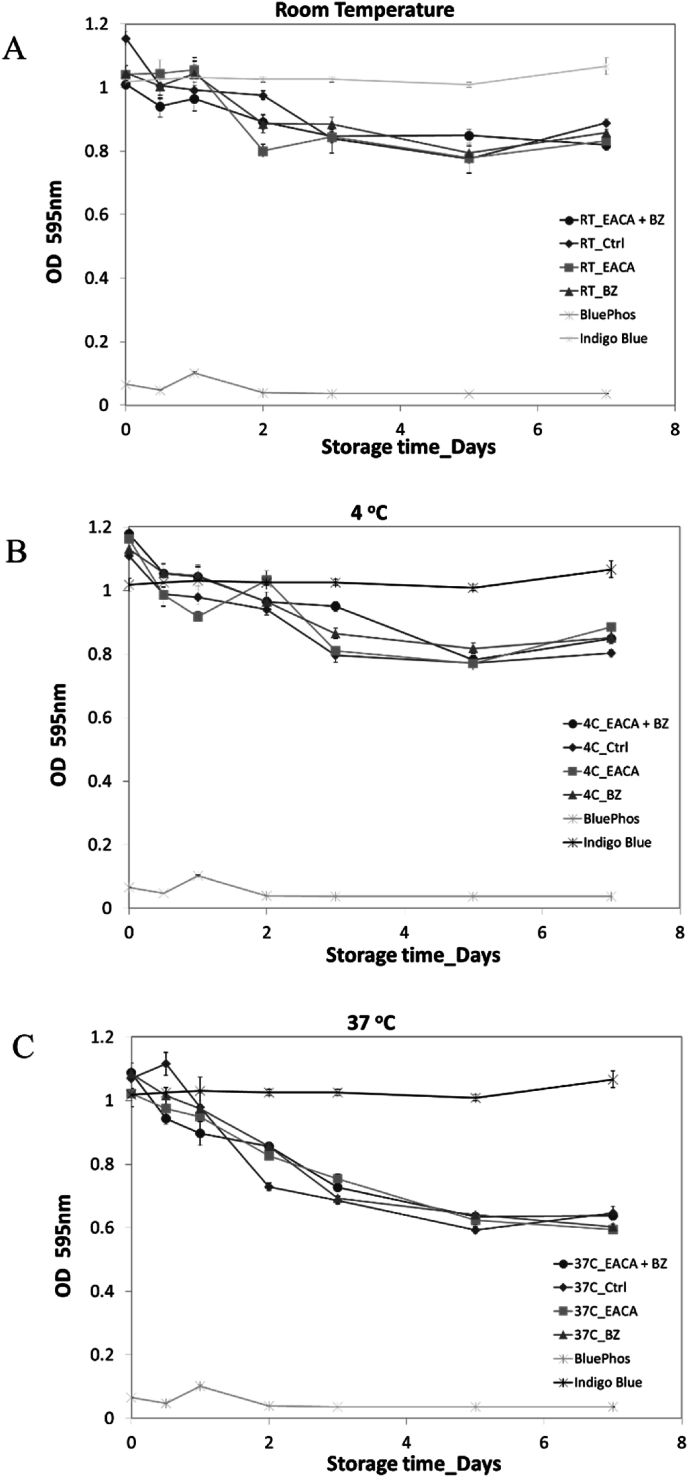
Scheduled sampling of the effect of storage temperature on APSA activity with and without the protease inhibitors. The effect of epsilon-aminocaproic acid (EACA) and/or benzamidine (BNZ) on liquid phase APSA activity over time on ice, at room temperature and at 37 °C is shown. Mean (n = 4) plus standard deviation shown. Panels: A, time course at room temperature; B, time course at 4 °C over 7 days; C, time course at 37 °C.

The effect of suicide and competitive inhibitors of serine centered protease on APSA activity were examined in 96 well plate assays. The APSA conjugate from 0 to 100 pg per well was added into the well. The wells were treated with control buffer versus covalent (suicide) protease inhibitors such as TLCK or AEBSF that cannot be washed off versus competitive protease inhibitors such as epsilon-aminocaproic acid (EACA) and benzamidine (BNZ) respectively. The suicide inhibitors TLCK and AEBSF completely abolished APSA activity and so were not suitable for testing a role for protease inhibitors in the preservation of APSA activity (Fig. 4). In separate experiments, 20 ng of APSA coated in 96 well plates was incubated with tris pH 8.85 or tris pH 8.85 plus 10 mM EACA or 10 mM benzamidine or the combination (n = 4) prior to measuring APSA activity (Fig. 5A) or washing six times in tris pH 8.85 prior to measuring the activity of the immobilized enzyme (Fig. 5B). The competitive inhibitors had no effect on the APSA activity. Next the competitive inhibitors were used to determine if APSA conjugate degradation by serine centered proteases plays any role in the loss of enzyme activity. Incubating immobilized APSA in 20 mM tris with or without the inhibitors or the combination had little protective effect on APSA activity over the course of 7 days on ice, at room temperature or at 37 °C (Fig. 6ABC). Thus, high storage temperature had a strong impact on the stability of the enzyme conjugate that seemed unrelated to tryptic or chymotryptic proteolysis.

### 3.5 The effect of freeze drying on APSA activity

**Fig. 7:**
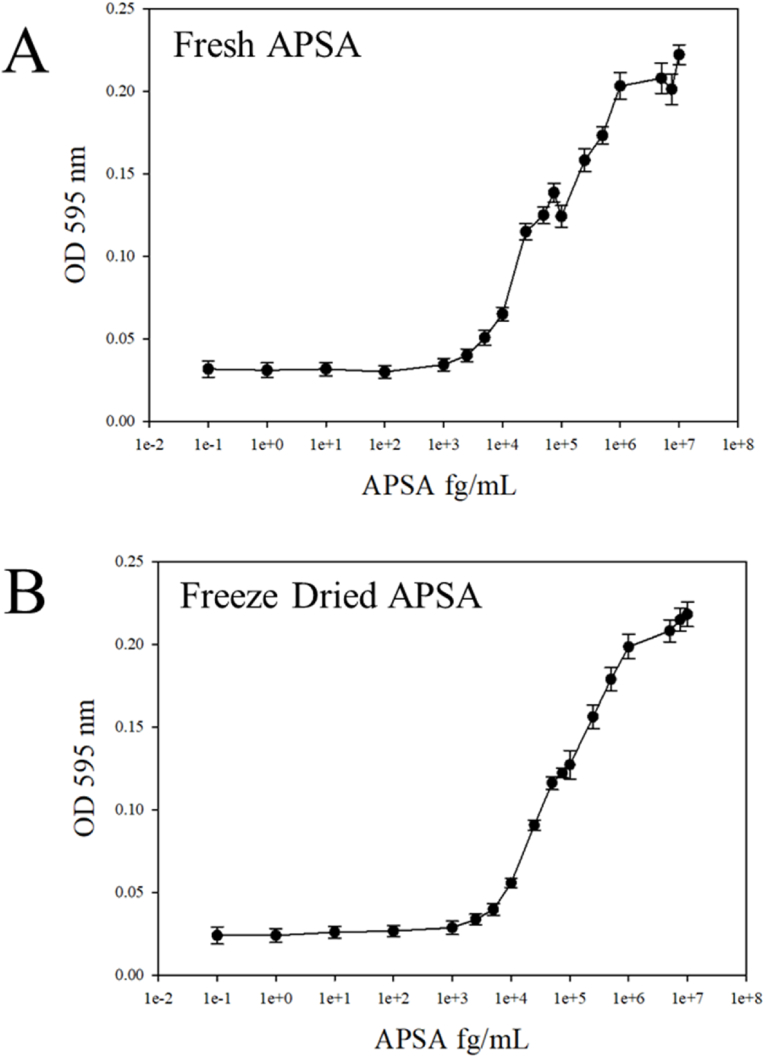
The effect of freeze drying on APSA activity. Fresh and freeze dried (24 h) APSA were titrated from the pico gram to nano gram per well immediately after freeze drying. Freeze dried APSA with a 1:1 ratio of 1 M sucrose and 1 M trehalose was resuspended and serially diluted in 20 mM tris HCl pH 8.85 at concentrations ranging from the femto gram (fg) to nano gram (ng) to reveal the detection limit. Panels: A, untreated control; B, freeze-dried in sucrose and trehalose. The samples were reacted with the BlusPhos dye and absorbance measured at 595 nm in a 96 well plate.

Freeze drying APSA in sucrose and trehalose had little immediate effect on APSA enzyme activity (Fig. 7AB). Thus, we did not observe that freeze drying itself had an immediate deleterious effect on APSA activity within 24 h of drying.

### 3.6 Standardization of liquid phase reaction APSA with indigo blue

**Fig. 8:**
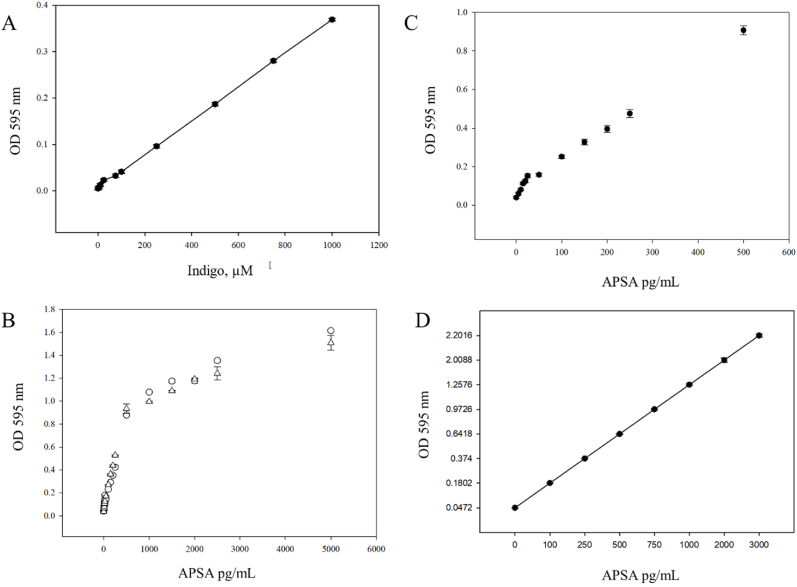
The standardization of the APSA enzyme conjugate by indigo blue in the Tween 20 enzyme reaction buffer for time of storage experiments. Panels: A, The dilution of the indigo blue in the reaction buffer with 1 % Tween 20 to serve as an analytical positive control at 1 OD unit is shown; B, titration of APSA conjugate to reach a starting OD 595 nm value of 1.0 [Symbols: (○) APSA sample one; APSA sample two (△)]; C The dilution of the APSA sample to yield 1 OD unit was established by UV/VIS analysis of the dilution curve; D, The broad linear range standard curve of APSA (100 pg/mL to 3 ng/mL) measured by the colorimetric BluePhos assay. Regression, Adjusted R^2^: 0.9933 F-statistic: 1629 on 1 and 10 DF, p-value: 2.088E-12. Mean (n = 4) 100 μL reactions plus standard error shown.

In order to make replicated measurements of APSA enzyme activity over long term storage conditions, a titration curve of the enzyme activity at the start of storage needed to be constructed for each sample against an indigo standard. Each vial of APSA may be characterized upon receipt by creating a titration curve that relates the concentration of the sample to OD 595 nm reading. The titration of APSA to reach an OD reading of 1.0 was determined at day zero (upon receipt). The APSA working concentration for each vial was defined as the concentration where an OD of 1.0 was achieved at 595 nm from BCIP/NBT substrate. An Indigo Blue absolute standard made up in DMSO served as a positive analytical control to account for day-to-day variation in the 96 well plate spectrometer (Fig. 8A). For enzyme assays the BCIP/NBT in reaction buffer alone served as a blank negative control. However different samples of APSA show different activity curves with a sharp linear slope between 0 and 100 pg/mL and a second gentler linear slope from ≥100 pg/mL (Fig. 8B,C&D). The indigo blue absolute standard made up in DMSO served as a positive analytical control to account for day-to-day variation in the 96-well plate spectrophotometer.

### 3.7 Effect of storage conditions on alkaline phosphatase activity over time

**Fig. 9:**
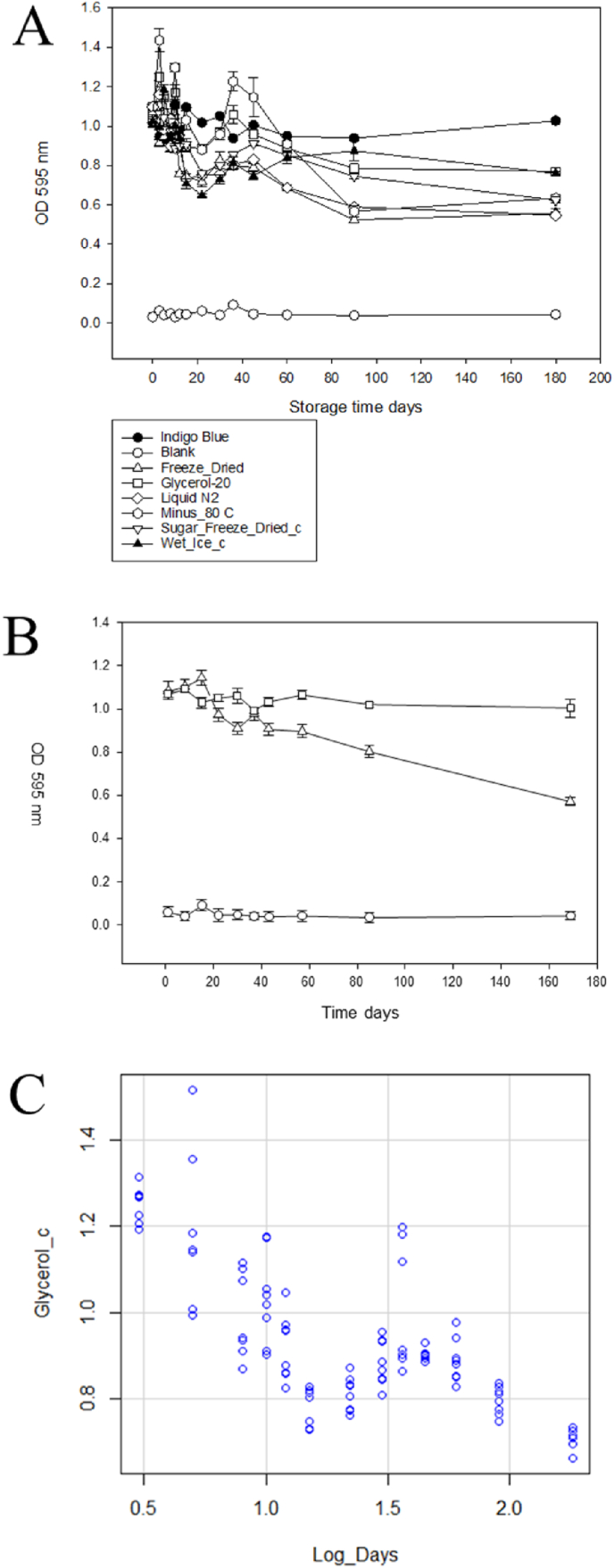
Comparison of the change of APSA's activity at different storage conditions over 25 weeks of storage. Panels: A, comparison of 6 different storage treatments and each mean represents 2 independent samples each with 4 technical replicates (n = 4); B, independent replication of the best result which is APSA in 50 % glycerol at −20 °C (△). The indigo blue dissolved in 10 % DMSO served as a positive control (□) [regression indicates that 200 μL of 840 μM indigo in 10 % DMSO is where OD = 1.0 at 595 nm]. The blank served as a negative control (○); C, regression of three independent samples of 50 % glycerol at −20 °C with three technical replicates contributing to each mean symbol shown (Adjusted R^2^: 0.5634, F-statistic: 133.9 on 1 and 102 DF, p-value: <2.2E-16). Mean (n = 4) plus standard error shown.

The variation in signal over samples indicated that in order to measure the degradation of APSA activity over time it will be necessary to titrate the sample at time zero so the recorded values over time will be within scale. The results of different storage conditions from the time-course study are shown in Fig. 9. Three separate samples of APSA stock ordered one month apart were titrated to determine the DF to reach OD 1.0 and original stocks aliquoted for long term storage experiments. The APSA enzyme activity was subsequently compared under six different storage conditions over a period of 170 days where each aliquot was exposed to no more than a single freeze-thaw cycle (Fig. 9A). The storage conditions compared were APSA on ice in the fridge (0–4 °C); in glycerol (1:1 v/v) in the freezer (−20 °C); freeze dried, and store in the freezer (−20 °C); APSA solution in the freezer (−80 °C); 1 M trehalose/1 M sucrose (1:1 v/v), freeze dried and stored in the freezer (−20 °C); APSA solution in liquid nitrogen. APSA stored on ice in a 4 °C refrigerator showed an alkaline phosphatase activity dropped from 100 % to less than 80 % initial activity in the first 3 weeks (Fig. 9A). The enzyme activity of APSA stored in 50 % glycerol at −20 °C showed a drop to 86 % of initial activity by week 6. APSA lyophilized without trehalose and sucrose seems to show greater variation from sample to sample but enzyme activity dropped decisively below 60 % of initial measurements by 10 weeks of storage. APSA freeze dried with sugars showed no immediate loss of activity after 24 h but with time dropped to about 50 % of starting activity after 10 weeks in storage. APSA stored at −80 °C was apparently concentrated by frost within the tubes and showed variation from aliquot to aliquot and some retained enzyme activity greater than 80 % until after 10 weeks of storage. The activity of APSA conjugate stored in liquid nitrogen dropped below 80 % within 3 week and eventually declined to 60 % of the starting value. A comparison of the APSA enzyme activity of the samples stored over time showed that fresh APSA showed the highest enzyme activity that dropped over the first 6–8 weeks as measured by the BCIP/NBT reaction alongside the Indigo Blue standard in DMSO. In an independent experiment, APSA conjugate was stored under the best treatment condition of 50 % glycerol and stored a −20 °C was compared to storage on ice that showed about a 20 % decline in enzyme activity in 3–6 weeks and eventually dropped to 60 % of the initial activity in over 10 weeks (Fig. 9B). The results with the independent sources of APSA showed very close agreement with the previous 50 % glycerol treatment at −20 °C and showed the good effect could be independently reproduced. The combination of the results from four independent samples of APSA in 50 % glycerol in −20 °C that showed a highly significant F statistic and a moderate R^2^ that seemed to show a decay of APSA enzyme activity over time where activity drops rapidly over the first 4–6 weeks and reached ∼50 % of starting activity after 170 days (F-statistic: 133.9 on 1 and 102 DF, p-value: <2.2E-16) (Fig. 9C).

## 4 Discussion

This study aimed to make a direct and comprehensive comparison of the effect of cold storage regime on APSA enzyme conjugate activity over time using entirely independent random sampling and scheduled sampling experiments that showed good agreement. The rate of APSA enzyme and/or binding activity loss was measured by random sampling of vials over different storage time duration and after titration to OD 1 showing similar results. The alkaline phosphatase-streptavidin (APSA) conjugate has both an enzyme catalytic and biotin binding activity that are both of central importance to many assays. Assays using selective binding reagents are an important application of enzyme conjugates that may suffer from variation in detection signal strength with serious health and economic impacts. The increased screening applications of binding reagents such as antibodies, proteins or binding domains has underlined the need for a complete and rigorous understanding in the storage and stability of the APSA enzyme conjugate [[17], [18], [19]]. It has been previously documented that the activity of alkaline phosphatase is sensitive to freeze drying, storage at room temperature or elevated temperatures [16,20,23]. A model system of biotinylated human IgG (B-h-IgG) was created for the purpose of comparing the detection limit of APSA over storage time. The results indicated that storing the APSA conjugate dry resulted in a large loss of activity that can be partially ameliorated by the addition of sugars but that ultra low temperature freezing such as −80 °C or liquid nitrogen was not helpful to maintain APSA activity over time.

### 4.1 Variation in APSA detection limits

The loss of sensitivity of the APSA conjugate in binding assays is a common confounding factor and source of irreproducibility in many biotin-based binding assays and showed a 20 % loss of activity over 4–6 weeks that may result in poor detection of the lower standards. The APSA conjugate showed large sample to sample variation in activity per unit volume. The variation in APSA binding and/or activity was a confounding factor in randomly sampling vials over time but showed the same trend and agreed with titration against indigo blue (Indigo Blue) followed by scheduled sampling over storage. The biotinylated human IgG (B-h-IgG) showed about a robust 10 ng detection limit on half sandwich solid phase PVDF and liquid phase reactions in half sandwich B-h-IgG bound to wells. Liquid phase half sandwich assays were linear from 1 to 100 ng IgG per well in 96 well plates. Thus, we conclude that the practical sensitivity limit of immobilized hIgG detection in a 96 well dish using only a biotinylated detector antibody is about 1–10 ng/well with the BCIP/NBT substrate reaction with APSA conjugate.

### 4.2 Crosslinking structural stability

The enzyme activity of alkaline phosphatase is only one component of the APSA conjugate and another activity is the binding property of streptavidin to biotin while a third feature is the glutaraldehyde cross linkage between the two proteins [29]. Temperature is a key factor that may affect the degradation but also conformation of proteins [16,20,23]. Recently it was shown that the dissolution of glutaraldehyde cross linked proteins structures may be significant over days in aqueous environment [30]. The capacity of the dot blot to efficiently localize the APSA signal to a 1-μL circular ring on the PVDF and to bind IgG immobilized on 96 well plates seem to indicate that the IgG binding function of streptavidin is robust over time in agreement with Western blots against the native conjugate that showed glutaraldehyde crosslinking [31] of the binding and enzymatic units are mostly stable. The signal from binding of the active alkaline phosphatase to the B-h-IgG immobilized in the 96 well plates over time showed a similar pattern to that of AP activity, indicated that the streptavidin remains bound for many months.

### 4.3 Conjugate degradation

The variation in the detection of the B-h-IgG analyte by the APSA conjugate may be accounted for in part by the loss of the enzyme activity that might result from oxidation, denaturation or proteolytic degradation. The modest proteolytic degradation observed by polyacrylamide electrophoresis was not concomitant with the significant loss of alkaline phosphatase activity overtime. The preparation apparently contains some active protease molecules and so storage in 50 % glycerol in the liquid state would be consistent with reducing protease activity over time without causing aggregation or inactivation from dehydration or ultra low freezing. Incubation at 37 °C resulted in a loss of half of the APSA enzyme activity over 7 days. However, the prediction and test of using competitive protease inhibitors BNZ and EACA had little protective effect of the APSA activity that ruled out a role for trypsin or chymotrypsin like proteases. The experiments were not able to rule out oxidation or denaturation of the conjugate over time.

### 4.4 Controlling analytical error with titration against an absolute indigo blue standard

Day to day variation in 96 well plate reading under different environmental conditions may also be a source of error that can be addressed using an absolute Indigo blue standard in DMSO. Here, the experiments were designed to measure the source of the variation in APSA detection limits over time that required determining the dilution factor for each sample to control the starting reaction OD value. It was possible to measure APSA against an indigo blue standard in DMSO setting to an OD value of 1.0 to correct the estimate of the loss of APSA function over the course of the seasons.

### 4.7 Freeze drying

Lyophilization can result in conformational changes leading to increased aggregation after reconstitution [24]. Enzymes are generally susceptible to chemical degradation and physical instability in aqueous solutions and freeze-drying (lyophilization) is a widely accepted strategy to retain satisfactory activity of enzymes during long-term storage [32]. The use of lyophilization (freeze drying) alone was not particularly successful method for preservation of APSA enzyme activity and resulted in 50 % of the starting OD 595 nm value within 4 months. The loss of activity may indicate that storing the APSA conjugate dry results in conformational changes over time.

### 4.5 Wet ice

The use of ice as a preservative is a viable option for use in shipping or in locations where freezers may not be available and was a simple and successful method to retain AP enzyme activity. In this study the APSA conjugate storage on wet ice was superior to all other methods except 50 % glycerol at −20 °C that was similar.

### 4.6 Storage in 50 % glycerol at −20 °C

Alkaline phosphatase activity is known to decrease in the presence of glycerol presumably from its effect on viscosity [4]. In general**,** storage in 50 % glycerol at −20 °C was the simplest method to preserved enzyme activity above 80 % of the initial OD value for the longest time. In particular, the 50 % glycerol treatment showed the best persistence of the enzyme activity, which corresponds to around 40 % loss of the activity during 120 days of storage. The protective effect of 50 % glycerol was consistent with previous observations of an increase in the conformational stability of proteins at a low temperature [12,13].

### 4.8 Freeze drying with trehalose and sucrose

Previously the combination of trehalose with other sugars has been shown to provide the best protection for the freeze drying of alkaline phosphatase alone [20,24]. Freeze drying is an option for transport and storage where ice, refrigeration or freezing is not available. Lyophilization with polyols such as sucrose and trehalose is an attractive option for storing biochemical samples and reagents. The drying process itself can impose unpredictable stresses on enzymes and subsequently lead to the conformational change or aggregation [24]. Therefore, additives such as trehalose and sucrose have been studied to preserve their activity at the solid state [33,34] consistent with the maintenance of 80 % of enzyme activity for 8 weeks compared to freeze drying without sugars. However, the important degradation mechanisms of proteins include aggregation, deamidation, oxidation and Maillard browning that may continue in the solid state [35,36]. Other reports have also indicated that drying with sugars prevented the conformational changes of the protein by embedding the protein molecules in the amorphous sugar matrices where structural water is replaced by sucrose and trehalose [[36], [37], [38]]. The observation of the best persistence in 50 % glycerol was consistent with the finding that other polyols such as sucrose and trehalose in the freeze dry medium were protective.

### 4.9 Ultra freezing at −80 °C

Temperature is a key factor that may affect degradation processes by limiting the mobility of the protein molecules. Putting APSA under ultralow temperature could be advantageous by reducing its motion, but freeze-thaw process may be damaging [39]. Storing APSA at −80 °C was a useful means to preserve the enzyme activity that was superior to lyophilization but resulted in less than 60 % of initial activity after 120 days storage.

### 4.10 Liquid nitrogen storage

Alkaline phosphatase is known to retain some enzyme activity even as low a −100 °C [40]. There are many studies on the maintenance of alkaline phosphatase activity in tissues, cells and biofluids under cryopreservation as a metric of viability [21]. However, there are few studies on the cryopreservation of APSA at the low temperature of liquid nitrogen (−195.8 °C) compared to other methods. Storage in liquid nitrogen apparently resulted in the loss of enzyme activity to about 50 % of the starting value after 120 days that may result from the freezing of structural water with denaturation of the enzyme.

## 5 Conclusion

Warm temperature storage (37 °C) has a severe deleterious effect on APSA conjugate activity. Two different independent sampling schemes agreed that enzyme activity of the APSA conjugate drops significantly over cold storage**.** Where freezers were available, APSA enzyme conjugate stored in 50 % glycerol at −20 °C was the simplest method. Where freezers are not available, wet ice was about as good as freezing at −20 °C in glycerol. Lyophilizing in trehalose plus sucrose showed some persistence of enzyme activity over a long-term storage versus freeze drying without sugars. Freezing in liquid nitrogen and lyophilization without sugars were the least useful methods. Protease inhibitors had little beneficial effect. Therefore, for practical purposes, and to obtain reproducible assay results, APSA should be shipped on ice or in 50 % glycerol at −20 °C. Similarly, it may be possible to avoid some negative effects by storing enzyme conjugate ice cold or at −20 °C with 50 % glycerol in the buffers. The variation in the APSA samples may result different detection limits between vials and over time that may be addressed in part by titration before use.

## Declaration of competing interest

The authors declare no conflict of interest.

## Data Availability

Data will be made available on request.
